# Racial Disparities in Surgical Management For Early‐Stage Laryngeal Squamous Cell Carcinoma and Recurrent Dysplasia

**DOI:** 10.1002/oto2.119

**Published:** 2024-02-28

**Authors:** Thomas F. Cyberski, Alexander Z. Wang, Brandon J. Baird

**Affiliations:** ^1^ Pritzker School of Medicine The University of Chicago Chicago Illinois USA; ^2^ Section of Otolaryngology–Head and Neck Surgery, Department of Surgery The University of Chicago Medicine Chicago Illinois USA

**Keywords:** cancer, disparities, dysplasia, larynx, race

## Abstract

**Objective:**

The aim of this study is to evaluate the association between race and the treatment of laryngeal dysplasia and early‐stage laryngeal squamous cell carcinoma (LSCC).

**Study design:**

Retrospective Cohort Study.

**Setting:**

Large multispecialty academic medical center.

**Methods:**

Patients were treated for laryngeal dysplasia or LSCC between September 2019 and September 2022. A retrospective chart review was conducted to collect demographic and clinical information. Two‐sample *t* tests, chi‐square tests, and linear regression models were used to compare characteristics (*α* = 0.05). Analyses were performed in STATA 17.

**Results:**

Sixty‐five patients were identified that underwent potassium titanyl phosphate (KTP) transoral laser microsurgery for management of early‐stage LSCC (n = 29) or dysplasia (n = 36). The cohort consisted of 23 Black and 42 White patients. No significant difference was found in age, alcohol or tobacco use, rate of adjuvant radiotherapy, stage of disease, nor insurance status between the 2 groups. White patients underwent more procedures to address initial disease and subsequent recurrent dysplasia on average than Black patients (2.52 vs 1.52, *P* = .02). This remained true after adjusting for demographic and clinical characteristics and insurance status in a linear regression model. While Black patients were more likely to be lost to follow‐up than White patients (30.4% vs 9.5%, *P* = .03), the average number of procedures between the groups still differed significantly (2.63 vs 1.56, *P* = .04) when controlling for those lost to follow‐up.

**Conclusion:**

The findings presented here highlight potential inequities that exist for racial minorities at early stages of treatment and in addressing premalignant conditions, which may contribute to the known downstream disparities in laryngeal cancer outcomes.

The larynx is one of the most common subsites of head and neck cancer, with an estimated incidence of 12,380 cases in 2023,^1^ and the vast majority of laryngeal cancers are laryngeal squamous cell carcinoma (LSCC).^2^ It has been shown that numerous sociodemographic factors contribute to disease morbidity and mortality in laryngeal cancer, including age, gender, income and education, insurance status, and race/ethnicity.^3^, ^4^, ^5^, ^6^ Black patients, in particular, have been demonstrated to have a worse laryngeal preservation rate than their White counterparts. They also have a higher mortality rate from laryngeal cancer overall.^7^, ^8^, ^9^, ^10^ While these disparities have been well documented, much of the literature on this topic is focused on outcome data in late‐stage disease.

Early diagnosis and prompt treatment is central to improving outcomes for patients with solid tumors, including head and neck cancer.^11^ While LSCC was formerly restricted to high morbidity treatment modalities, like total laryngectomy for late‐stage disease, the increasing utility of transoral laser microsurgery (TLM), has made earlier, less‐invasive surgical treatment options possible.^12^ TLM has been demonstrated to increase laryngeal preservation rates in patients with early‐stage LSCC relative to treatment with upfront radiotherapy, without significant functional sequelae.^13^ Some patients who have completed treatment for early‐stage LSCC or severe dysplasia demonstrate recurrent leukoplakia that is ultimately determined to be recurrent/refractory dysplasia. If these lesions interfere with mucosal wave, cause dysphonia, or increase in footprint, they can be addressed using selective potassium titanyl phosphate (KTP) laser photoangiolysis to decrease risk of progression to malignancy, diminish the effect of the leukoplakia on mucosal wave propagation, and help to maintain good functional voicing.^14^

Although much work has been done to highlight the disparities that exist in laryngeal cancer outcomes for racial minorities, it is important to investigate access to care at various stages of treatment, especially as improved treatment methods are developed, to better assess where disparities are occurring and identify ways they can be remedied. Thus, the aim of this study is to better understand the patterns of recurrence and treatment of recurrent laryngeal dysplasia and identify inequities in care that may exist after an initial surgical laryngeal preservation strategy for LSCC and pre‐malignant pathology.

## Methods

A retrospective chart review was conducted to investigate characteristics of patients treated for laryngeal dysplasia or LSCC at a large multispecialty academic medical center. Patients were identified for initial inclusion in the study based on having an operative report for TLM between September 2019 and September 2022, who subsequently followed for dysplasia/early glottic cancer surveillance. Those that required office‐based KTP laser surgery (OLS)/TLM or repeat OLS/TLM prior to 2019 were included in the analysis as well. Patients who underwent TLM for any diagnoses other than laryngeal dysplasia or LSCC were excluded. Upon establishing the final study cohort, demographic and clinical information were collected for each patient. Characteristics evaluated included age, gender, race, and ethnicity (as self‐identified by patients within the electronic medical record), risk factors, histopathology, treatment modality, number of procedures required to address disease (including both operative via TLM and office‐based via office‐based KTP laser surgery [OLS]), adjuvant radiation therapy, disease recurrence, insurance status (private, public, or uninsured), and mortality. Ethnicity was defined as Hispanic or Latino or non‐Hispanic or non‐Latino. As noted by the NIH, “people who identify their origin as Hispanic, Latino, or Spanish may be of any race,”^15^ so race and ethnicity were characterized separately. Most of the procedures undergone by the study cohort were conducted by 3 fellowship‐trained laryngologists at 2 different institutions, however prior OLS/TLM procedures conducted by providers at outside institutions were included in analysis as well.

The cohort was stratified by racial status for further statistical analysis. Two‐sample *t* tests, chi‐square tests of independence, and linear regression models were used to examine differences between the groups. Patients were determined to be lost to follow‐up if they did not attend their scheduled return visits postoperatively and length of follow‐up was calculated from the date of the patient's initial surgery to their most recently attended visit. All analyses were performed in STATA 17.0 (StataCorp, 2021), with a *P* < .05 determined as significant. The institutional review board at the University of Chicago (IRB21‐1864) approved this study.

## Results

Sixty‐five patients were identified that underwent TLM for management of early‐stage LSCC or dysplasia during the study period, of which, 23 of the patients were Black (35.4%) and 42 were White (64.6%). The ethnic status of the vast majority of the cohort was non‐Hispanic (n = 62, 95.4%). Only 2 patients identified as Hispanic or Latino (1 Black patient and 1 White patient) and 1 as unknown ethnicity (Black patient), leaving too few patients for subgroup analysis by ethnicity. Black and White patient subgroups were not found to differ significantly in mean age (68.2 vs 66.4, *P* = .511), but were found to differ significantly in their proportion of female patients (30.4% of Black patients vs 7.1% of White patients, *P* = .013) (Table 1). Both groups had similar rates and types of insurance coverage. Ten out of 23 Black patients had private insurance (43.5%) and 13 had public insurance (56.5%). Twenty‐two out of 42 White patients had private insurance (52.4%), 19 had public insurance (45.2%), and 1 was uninsured (2.4%). Insurance status did not differ significantly between Black and White patients (*P* = .56).

**Table 1:** Racial Subgroup Demographic Characteristics

	Black patients (N = 23)	White patients (N = 42)	*P* value
Age, mean (SD)	68.2 (2.18)	66.4 (1.61)	.511
Female gender, No. (%)	7 (30.4)	3 (7.14)	.013*
Ethnicity, No. (%)			
Hispanic	1 (4.35)	1 (2.38)	.354
Risk factors, No. (%)			
Tobacco use	21 (91.3)	32 (76.2)	.133
Alcohol use	13 (56.5)	27 (64.3)	.538

* Statistically significant.

Both groups also had similar rates of reported tobacco and alcohol use, with 91.3% of Black patients being current or former smokers compared to 76.2% of White patients (*P* = .133) and 56.5% Black patients had a history of alcohol use compared to 64.3% of White patients (*P* = .538). Of the patients with a history of smoking, the average amount of pack years smoked per patient did not differ significantly between Black and White patients (33.5 pack years vs 33.7 pack years, *P* = .973). Among Black patients, 52.1% were treated for dysplasia/carcinoma in situ (CIS), with the other 48.8% receiving treatment for LSCC (Table 2). Similarly, 57.1% of White patients were treated for dysplasia/CIS and 42.9% for LSCC (*P* = .700). Of those treated for dysplasia/CIS, the majority had severe/CIS grade lesions (58.3% of Black patients vs 66.7% of White patients, *P* = .826). The most common laryngeal subsite of disease for both Black and White patients was the glottis, at 95.7% and 95.2% respectively (*P* = .939). Only 1 out of 23 Black patients (4.35%) and 2 out of 42 White patients (4.76%) had supraglottic disease (Table 2).

**Table 2:** Racial Subgroup Clinical Characteristics

	Black patients (N = 23)	White patients (N = 42)	*P* value
Histopathology, No. (%)			
Dysplasia/CIS	12 (52.2)	24 (57.1)	.700
LSCC	11 (57.8)	18 (42.9)	
Dysplasia grade, No. (%)			
Mild	4 (33.3)	7 (29.2)	.826
Moderate	1 (8.33)	1 (4.17)	
Severe/CIS	7 (58.3)	16 (66.7)	
Laryngeal subsite, No. (%)			
Supraglottis	1 (4.35)	2 (4.76)	.939
Glottis	22 (95.7)	40 (95.2)	
Outcomes, No. (%)			
Adjuvant RT	4 (17.4)	10 (23.8)	.547
Tracheotomy	0 (0.0)	2 (4.76)	.288

Abbreviations: CIS, carcinoma in situ; LSCC, laryngeal squamous cell carcinoma; RT, radiation therapy.

**Figure 1:**
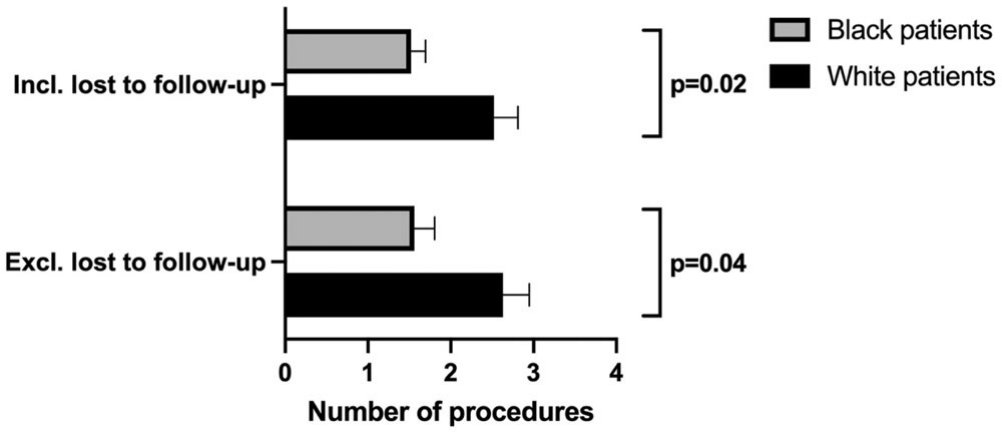
Bar graph depiction of the average number of procedures per patient in each racial subgroup before and after controlling for those lost to follow‐up. Error bars represent standard error.

**Figure 2:**
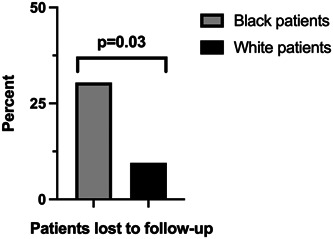
Bar graph depiction of the percentage of patients lost to follow‐up after transoral laser microsurgery in each racial subgroup.

Upon treatment of their disease, including both operative TLM and prior or subsequent OLS, Black patients ultimately underwent significantly fewer procedures on average than White patients (1.52 vs 2.52, *P* = .019), with only 34.8% of Black patients receiving 2 or more procedures compared to 64.3% of White patients (Figure 1). Black patients were found to be more likely to be lost to follow‐up than White patients, with 30.4% and 9.5% lost to follow‐up in each group respectively (*P* = .032) (Figure 2). After controlling for those lost to follow‐up, the average length of follow‐up did not differ significantly between groups, with Black patients following up on average for 66.2 weeks compared to 82.0 weeks for White patients (*P* = .330). The average number of procedures between the groups still remained significantly different when controlling for those lost to follow‐up. Black patients on average received 1.56 procedures compared to 2.63 procedures on average for White patients (*P* = .043). In our linear regression model, we found that Black patients received fewer procedures than White patients, even after adjusting for sex, age, race, pathology subtype, disease site, and insurance status (*P* = .043).

Black and White patients required adjuvant radiotherapy for recurrent disease at similar rates (17.4% vs 23.8%, *P* = .547). Because the study population was made up of patients recently treated for laryngeal dysplasia and early‐stage LSCC, long‐term outcome data for this cohort was limited. Only 2 patients (3.1%) from the cohort have required a tracheotomy—both of which were not related to progressive/recurrent LSCC disease. One patient underwent a tracheotomy at an outside hospital due to post‐operative airway obstruction secondary to an enlarging base of tongue hematoma after undergoing TLM for LSCC and the other patient had a postoperative hemorrhage following the resection of a synchronous primary cancer of the tongue, unrelated to the patient's laryngeal disease. Thus far, none of the patients in the cohort have subsequently undergone a total laryngectomy. Overall, the cohort has a 0% disease‐specific mortality rate and a 1.5% all‐cause mortality rate.

## Discussion

In this retrospective study, Black patients received TLM for early‐stage treatment of LSCC and OLS for recurrent dysplasia at a significantly lower rate than White patients, despite the 2 groups having similar risk factors, insurance status, and levels of disease. Our study adds to the existing literature by highlighting another point in the treatment process where racial disparities in care may be occurring. This finding is consistent with previous work showing that Black patients are more likely to receive total laryngectomies for earlier‐stage disease and less likely to receive local, endoscopic surgery for early‐stage disease management.^7^, ^8^ Together, these findings highlight the need for future work that critically evaluates each step of the decision‐making process in the treatment of laryngeal cancer.

We also found Black patients were significantly more likely to be lost to follow‐up than White patients. While this was not our primary outcome, this finding supports previous findings showing disparity in access to early laryngeal cancer care related to race.^16^ However, even after controlling for patients lost to follow‐up, Black patients still underwent fewer procedures to address recurrent dysplasia than their White counterparts.

We also found no significant difference in disease‐specific mortality rate by race in our cohort. Previous work has suggested that racial disparities in LSCC survival may be related to differences in disease burden at presentation.^17^ As both White and Black patients presented with similar levels of disease burden at time of diagnosis, the health disparity that exists (Black patients having more advanced disease) may be more representative of differences in patterns of follow‐up for this cohort, rather than inherently more aggressive pathology at time of presentation.

One might hypothesize that the increased number of procedures for White patients is representative of a more refractory, recurrent dysplasia that requires more frequent intervention. However, these discrepancies may, instead, reflect a lack of access to health care, more advanced disease at presentation or higher degree of response to selective photoangiolysis when comparing the 2 groups. These results are consistent with previous research showing that Black patients are more likely to present with late‐stage laryngeal cancer and less likely to have laryngeal preservation when compared to White patients.^7^, ^10^ By increasing access and opportunity for Black patients to receive OLS and TLM, subsequent disparities in management of advanced disease may diminish.

Health inequities in laryngeal cancer care exist for a number of reasons. Insurance status and coverage is a frequently discussed explanation for disparities in laryngeal cancer care. Ethnic and racial minorities often have higher rates of uninsured than non‐Hispanic, White patients.^18^, ^19^ Studies have found that patients that are uninsured or covered by Medicaid are at higher risk of presenting with more advanced laryngeal cancer, higher mortality rates, and greater odds of receiving total laryngectomy than patients with private insurance.^20^, ^21^, ^22^ Other studies have suggested that insurance status may serve as a proxy for other medical care access issues, such as regular screening and preventative appointments.^23^ The importance of accessible medical care is highlighted by previous work that found no difference in LSCC mortality between Black and White patients treated within the Veterans Health Administration, while a difference in overall survival was observed in patients evaluated using the Surveillance, Epidemiology, and End Results (SEER) database.^17^ These results are telling, as the SEER database is more representative of the diverse insurance landscape within the US health care system. Black patients are significantly more likely to have unstable insurance status than White patients, which may negatively impact their ability to be referred to a specialist.^24^ Together, these previous studies suggest the presence of a delay or lack of referral for endoscopic management of early‐stage disease amongst Black patients. This, in turn, may lead to subsequent progression of disease, requiring a higher rate of upfront total laryngectomy to eradicate disease. We are not able to definitively say if this was the case for our cohort, as we were unable to track long‐term insurance stability and our cohort contained a limited amount of uninsured patients. Thus, this may be an important topic of future investigation in this population of patients. Additionally, there was no significant difference in tumor stage at time of presentation, suggesting that Black and White patients presented with equivalent burden of disease.

Disparities in health literacy may be another reason for the discrepancy in number of procedures performed for patients with recurrent dysplasia. Previous studies have suggested that more than one‐third of patients with head and neck cancer may have severely inadequate health literacy.^25^ In particular, patients from racial or ethnic minority groups and patients with lower socioeconomic status were found to be more likely to have lower or inadequate health literacy.^26^ As a result, these patients may delay or not seek medical care and struggle to understand the health information they are presented.^26^, ^27^ Moreover, Black patients are more likely to receive radiotherapy and less likely to be offered surgery as a treatment option for laryngeal cancer treatment.^8^, ^28^ Thus, we speculate that options to pursue OLS/TLM may be limited and/or patients that are candidates may not always be aware of said options. This might account for why Black patients in this study were less likely to receive repeat TLM or OLS than White patients.

The previously proposed explanations for our findings hint at the idea that better socioeconomic status results in better outcomes for laryngeal cancer. Factors such as insurance status, access to medical care, and health literacy are all characteristics that reflect this disparity. While the previously described explanations are supported by the existing literature, they may not tell the full story. Black patients continue to have worse 5‐year overall survival for laryngeal cancer than White patients across all neighborhood socioeconomic status and income levels.^18^, ^29^ Another reason that we see a disparity between Black and White patients may be a result of mistrust of the medical system due to historical trauma and racism.^29^ This may result in Black patients being less likely to return for repeat TLM or OLS procedures. While systemic racism and health inequity may play a role here, it is unclear how exactly inequity in access to TLM and OLS may affect downstream health disparities in laryngeal cancer care. We recognize that racial disparities in health care are often multifactorial, including but not limited to income, education, and other medical comorbidities. Future research is needed to better understand how each of these factors affect the racial disparities that exist in the treatment of laryngeal cancer.

There are limitations to our study including small sample size and the fact that the study was retrospective in nature. All members of the cohort were treated at an academic medical center or academic‐affiliated sites in a large, metropolitan area by 3 different fellowship‐trained laryngologists. Differences in practice setting may change these findings. Additionally, discrepancies in practice pattern (how recurrent dysplasia is addressed) might have a variable impact on the total number of procedures offered, as well as short‐ and long‐term outcomes. Another limitation may stem from our definition of “lost to follow up.” We recognize that our definition does not capture patients that sought post‐operative treatment at a different institution. Future prospective studies may be needed to better characterize this nuance in the surgical treatment of recurrent dysplasia. The low numbers of female and Hispanic/Latinx patients could potentially limit the generalizability of these findings. We are also unable to control for differences in insurance status and health literacy. Future prospective studies are needed to further investigate the effect of addressing racial disparities in early‐stage laryngeal cancer care and management of dysplasia on laryngeal cancer outcomes. More research into how these factors contribute to racial disparities in treatment of LSCC and utility of office‐based laser surgery are needed.

## Conclusion

In conclusion, this study demonstrates that Black patients undergo TLM/OLS less frequently for early‐stage LSCC treatment than White patients, irrespective of follow‐up status. These results are consistent with existing literature about racial disparities in treatment and outcomes for laryngeal cancers. We hope that addressing racial disparities in early‐stage laryngeal cancer and pre‐malignant conditions may help alleviate more downstream disparities in treatment and outcome.

## Author Contributions

**Thomas F. Cyberski**, design, conduct, analysis, and presentation; **Alexander Z. Wang**, conduct, analysis, and presentation; **Brandon J. Baird**, design, analysis, and presentation.

## Disclosures

### Competing Interest

Burroughs‐Wellcome Fund (TFC).

### Funding source

Burroughs‐Wellcome Fund (TFC).
